# Barriers and facilitators of mental health app use among young women – a multimethod study

**DOI:** 10.1186/s12905-026-04934-w

**Published:** 2026-09-26

**Authors:** Johanna Schröder, Sandra Miethe, Luana López, Alina Emde, Sebastian Trautmann

**Affiliations:** 1 Institute for Clinical Psychology and Psychotherapy, Department for Psychology, Medical School Hamburg - University of Applied Sciences and Medical University, Am Kaiserkai 1, Hamburg, 20457 Germany; 2 Nalei GbR, Hamburg, Germany

**Keywords:** Distress, Young women, Help seeking, Mental health app

## Abstract

**Background:**

Prevalences of psychological distress among young women are high and rising. Despite a vast number of potentially helpful mental health apps (MHA), dissemination is limited. The current study aims to inform user-centered development and implementation of mental health apps for young women by examining mental health problems in the target population, existing help-seeking patterns and their gaps, perceived barriers to both conventional and digital care, and preferred MHA characteristics.

**Methods:**

521 women between the ages of 18 and 35 participated in a cross-sectional online survey. Alongside descriptive data on psychological distress, help-seeking behaviour, and barriers to mental health app use, associations between indicators of psychological distress and MHA use were examined using binary logistic regression. Thematic analysis was used to reveal attributes of MHA that are considered professional and most helpful by this group.

**Results:**

The most common psychological distress symptoms were fatigue (79%), worry (79%), and tension (74%). Frequently used support sources included self-help books (60%), primary care (51%), and psychotherapy (51%); 31% had ever used MHA. Barriers to MHA use were mainly a lack of knowledge (32.5%), concerns about effectiveness (9.8%), and financial costs (9.6%). General psychological distress (OR = 1.03 [1.01–1.06], *p* = .012), anxiety and appetite/weight problems (ORs between 1.95 and 1.99, *p*s ≤ .004) were most strongly associated with MHA use. Thematic analysis showed that young women view professional appearance and helpfulness in MHA as multifaceted, emphasizing usability, security, as well as personal and technical support as relevant aspects.

**Conclusions:**

Although psychological distress is common among young women, only a minority uses MHA. Use is associated with the type of symptoms, symptom burden, knowledge of and expectations toward these tools. Improving awareness and accessibility, ensuring app quality, credibility, and data security may represent key factors for strengthening uptake and sustained use in this population.

**Supplementary Information:**

The online version contains supplementary material available at https://doi.org/10.1186/s12905-026-04934-w.

## Introduction

The Global Burden of Disease study showed a high burden of internalizing disorders (e.g., depressive disorders, anxiety disorders, and eating disorders), especially in women [1]. In younger age cohorts, the prevalence of anxiety and depression increased substantially between 2008 and 2013 for girls and to a lesser extent for boys [2]. This same study concludes that especially girls and young individuals with poor social support experience mental health problems. Social support appears to have a stronger role as a protective factor for psychological distress among young women, compared to young men and older women and men [3]. A recent systematic review on help-seeking behaviour [4] showed that female adolescents tended to have higher mental health literacy and more adaptive attitudes regarding mental health problems, including greater help-seeking knowledge and intentions. A study using an Australian sample aged 16 to 24 years [5], found that young women were more inclined than young men to uptake app usage, particularly those designed to improve health and well-being. In contrast, young men showed greater interest in gamification features such as tracking and tended to lose interest in such apps more quickly. Against this background, the present study reports findings from a sample that was predominantly female, which reflects the composition of the recruited sample rather than an a priori design decision, but which aligns with the documented tendency of female adolescents to engage more readily with digital mental health interventions. Engagement with digital mental health interventions is commonly examined through the related concepts of usability and adherence [6], that is, the ease with which users can interact with an application and the degree to which users engage with the program.

Mental health apps (MHA) represent a conceptually and functionally distinct category within the broader landscape of digital mental health interventions and have significant potential to deliver high-efficacy mental health interventions and bridge the mental health treatment gap [7]. Unlike browser-based programs, apps installed on mobile phones enable ubiquitous access. Apps enable features such as push notifications, passive sensing, and ecological momentary assessment that are technically and functionally distinct from browser-based platforms [8] and carry specific implications for engagement and implementation. Evidence suggests that the delivery platform itself influences engagement patterns: compared to web-based delivery, app-based delivery is associated with more frequent logins, broader use of available intervention components, and shorter usage sessions [9]. Results from an umbrella review [10] summarised the key potential of MHA in helping to provide timely support, ease the costs of mental healthcare, combat stigma in help-seeking, and enhance therapeutic outcomes. Challenges include adherence issues, safety concerns in emergencies, privacy and confidentiality breaches, and the use of non-evidence-based approaches. A meta-analysis [11] emphasized that low utilisation of digital mental health interventions remains a key challenge, substantially limiting their overall impact.

With this background, the potential to improve mental health among young women through MHA can be expected as substantial. It is therefore important to understand relevant factors for health service use and technology use in this population. Andersen’s behavioural model for health services utilization [12] explains health care use as a function of predisposing factors existing prior to illness (e.g., demographics and knowledge or attitudes regarding health and health care services), enabling factors like personal or community services (e.g., income, service accessibility), and need factors (e.g., perceived and professionally evaluated need). The *Extended Unified Theory of Acceptance and Use of Technology* [13] explains individuals’ use intention and use of technology as driven less by objective features of a technology than by users’ expectations about its usefulness, ease, cost, and social endorsement. Shedding light on circumstances, expectations, motivations, and barriers of young women regarding MHA utilization may help developers and mental health professionals design and disseminate mental health apps that enhance adoption of professional interventions and, in turn, reduce the mental health burden among young women. The current study therefore aims to address the following questions: (a) how do young women experience psychological distress, defined as a broad concept characterised by unpleasant feelings or emotions that people may experience as overwhelming [14]; (b) how do they seek help in times of psychological distress; (c) what are probabilities and predictors of MHA use; (d) which are barriers they perceive regarding MHA use; (e) which elements of MHA do they perceive as professional; and (f) which elements of MHA do they perceive as most helpful. Generating knowledge in this area contributes to characterizing adoption, which is a prerequisite for deriving user-centered design recommendations in accordance with person-based approaches to intervention development [15].

## Materials and methods

### Study design and procedure

The cross-sectional online study was conducted in compliance with the Declaration of Helsinki [16] and approved by the local ethics committee of Medical School Hamburg (reference number MSH-2023/237). Based on a cross-sectional anonymous online study in the period from March 2023 to May 2023, individuals aged 18 to 35 years were invited to participate in the survey. The survey, which was programmed with the software ‘efs survey/unipark’, consisted of a minimum of 38 and a maximum of 40 single or multiple-choice items and open-ended questions with text fields. Digital informed consent was required before starting the online survey. Violating the inclusion criterion led to termination of the online assessment, whereupon the participant was informed of the reason for their exclusion.

### Participants and recruitment

The inclusion criterion was an age between 18 and 35 years at the time of the survey. Recruitment of the convenience sample was conducted online via German-speaking social media (LinkedIn and Instagram) and the student e-mail distribution list of Medical School Hamburg. Although the study originally aimed to include participants of all genders, a very small percentage of male (*n* = 28) and diverse (*n* = 3) participants led to the decision to focus exclusively on female participants.

### Measures

The participants provided sociodemographic (age, relationship status, parenthood, income after taxes) and clinical information (psychological distress in the last four weeks, lifetime diagnoses of mental disorders). Psychological distress in the last four weeks was assessed using the ten-item, self-report measure *Kessler Psychological Distress Scale* (K-10) [14], which is a global routine measure of distress based on questions about anxiety and depression symptoms. The internal consistency in the current sample was α = 0.91. To assess how the participants experience psychological distress, they were invited to answer the question “How does psychological distress typically manifest in your experience?“ using a checkbox list of 15 typical distress symptoms and behaviours (yes/no; multiple answers possible). To assess help-seeking behaviour, the participants were asked “Have you ever used any of the following forms of support due to psychological distress?”, accompanied by a checkbox list of eleven clinical and psychosocial support and self-help services and the three answering options ‘no, never’, ‘yes, once but not currently’, and ‘yes, currently’. Only those participants who indicated never having used MHA were led to the question “What has prevented you from using a mental health app when experiencing psychological distress?”, accompanied by an open text box field. Lifetime use referred to whether a participant has ever used an MHA at any point in their life, irrespective of current or continued use. Current use referred to active or ongoing use of an MHA at the time of assessment. Online interventions referred to Internet-delivered, often browser-based, guided or unguided mental health programs. Further, two questions assessed the young women’s attitudes towards MHA, again using open text fields: “How do you recognize professionalism in a mental health app?”; “What would the most helpful mental health app offer?”. The participants were provided with the following definition of MHA: “software applications designed to be downloaded and used on mobile devices for the purpose of supporting, maintaining, or improving mental health“, before answering the relevant questions.

### Data analysis

Associations between indicators of psychological distress and MHA use (lifetime or current) were examined using simple and multiple binary logistic regressions. To be able to evaluate potential collinearity in the multiple regression models, a correlation matrix of the psychological distress predictors is provided in the supplementary material [see Additional file 1]. Variance Inflation Factors of the predictors varied between 1.06 and 1.30, providing no indication of substantial multicollinearity. Odds ratios (OR) with 95% confidence intervals (CI) are reported to quantify associations. Statistical significance was evaluated two-sided at the 5% threshold. The text material was analysed by one coder using reflexive thematic analysis according to Braun and Clarke [17], which is a qualitative research method for identifying patterns in text data. The material was analysed by a psychology student (LL). After becoming familiar with the data and gaining an understanding for the content, initial themes were generated based on text segments that were meaningful with regard to the research questions. The organization of themes and subthemes was not predetermined but emerged inductively through iterative engagement with the data, reflecting the researchers’ interpretive position rather than an a priori structural framework. In a second coding, run the themes were refined and validated by ensuring that they accurately reflected the content of the data. Once the data had been analysed, the topics were thematically clustered into themes, which are patterns of shared meaning underpinned by a central organizing concept. Ambiguous cases and decisions regarding theme development and interpretation were solved through ongoing reflection and discussion within the research team. The sample of individuals who responded to the respective question served as a reference for the percentages.

## Results

### Sample characteristics, psychological distress symptoms, and help-seeking behaviour

Overall, 521 women at an age between 18 and 35 (*M* = 24.7, *SD* = 3.8) completed the online survey. Only 4.8% of the sample showed no clinically relevant psychological distress, 22.5% reported mild distress, 37.8% reported moderate distress, and 34.9% reported severe psychological distress. Further, 44% reported having received a diagnosis of a mental health disorder at some point in their lifetime (see Table 1).

**Table 1:** Sample characteristics

	*N* = 521
Age, M (SD)	24.7 (3.8)
Partnership status, *n* (%)	
Single	185 (35.5)
Intimate relationship	336 (64.5)
Parenthood	
Yes	24 (4.6)
No	497 (95.4)
Income after taxes, *n* (%)	
< 500€	135 (25.9)
> 500–1000€	122 (23.4)
> 1000–1800€	94 (18.0)
> 1800–2500€	113 (21.7)
> 2500–3000€	38 (7.3)
> 3000€	19 (3.7)
Psychological distress (K-10), *n* (%)	
None	25 (4.8)
Mild	117 (22.5)
Moderate	197 (37.8)
Severe	182 (34.9)
Self-reported diagnosed mental disorder (lifetime), *n* (%)	
Yes	229 (44.0)
No	292 (56.0)

The most frequently reported symptoms, each reported in more than 70% of the sample, were fatigue/exhaustion, worries/brooding, and tension (see Table 2).

**Table 2:** Expression of psychological distress in the sample of young women (*N* = 521)

	%
Tension	73.7
Anxiety	64.9
Depressed mood	58.4
Sadness	56.4
Irritability	62.4
Headaches	41.3
Fatigue/Exhaustion	79.3
Worries/Brooding	78.7
Social conflicts	24.4
Social withdrawal	53.6
Sleeping problems	48.9
Appetite or weight problems	31.1
Stomach/intestinal problems	39.5
Concentration problems	50.5
Substance use	9.4
Other	7.3

Multiple answers were possible

**Fig. 1:**
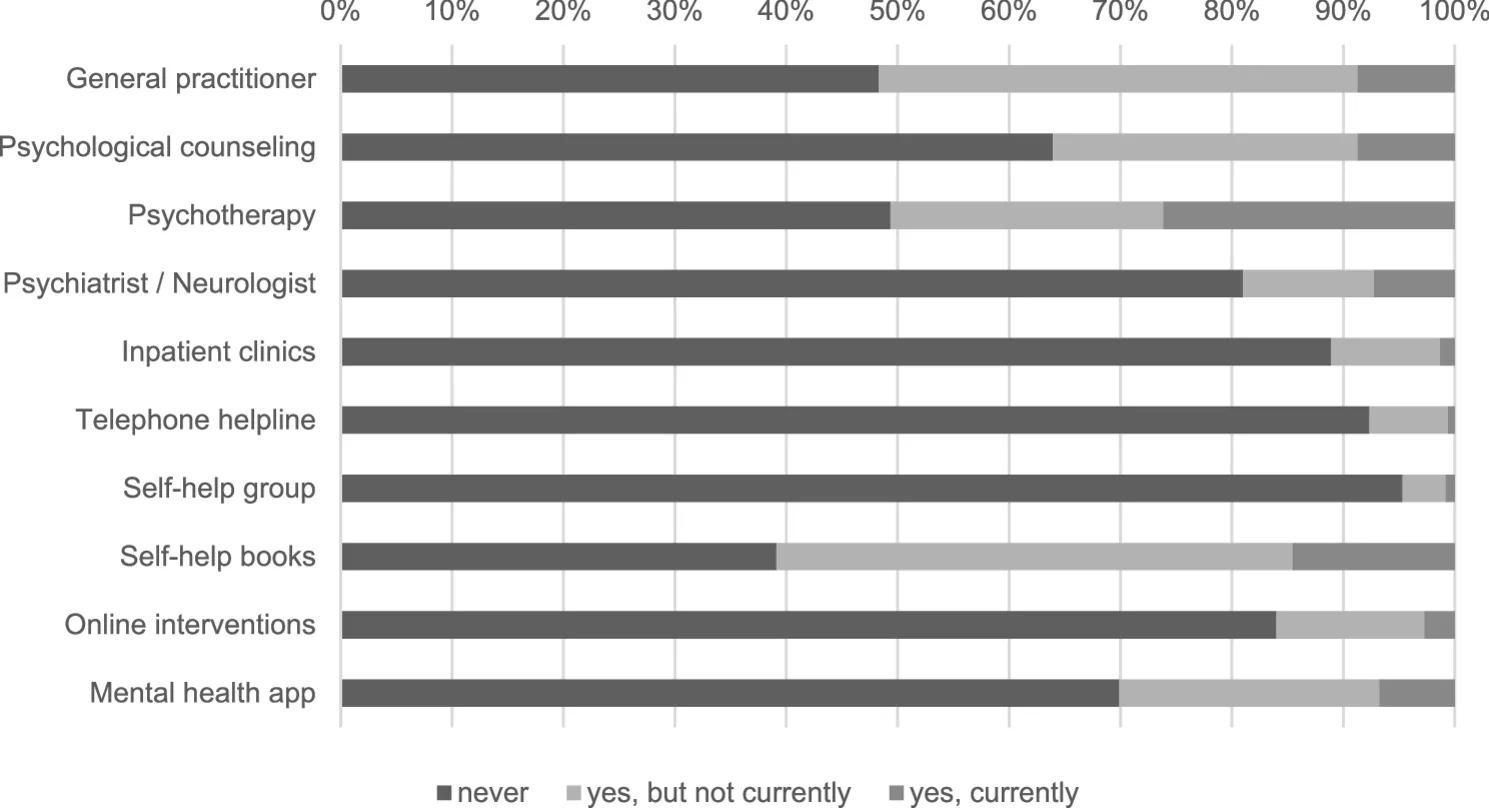
Help-seeking behaviour in times of psychological distress in the sample of young women (*N* = 521)

The most frequently used support modalities in the lifetime of the participants were self-help books (60.3%), psychotherapy (51.3%), and primary care (51.3%). The least frequently used support modalities or structures were self-help groups (6.3%) , telephone helplines (9.4%), and inpatient clinics (12.5%). The use of online interventions at some point in their life was reported by 17.1% of the sample. Lifetime use of mental health apps was reported by 31.1%, while 6.7% reported current use (see Fig. 1).

**Fig. 2:**
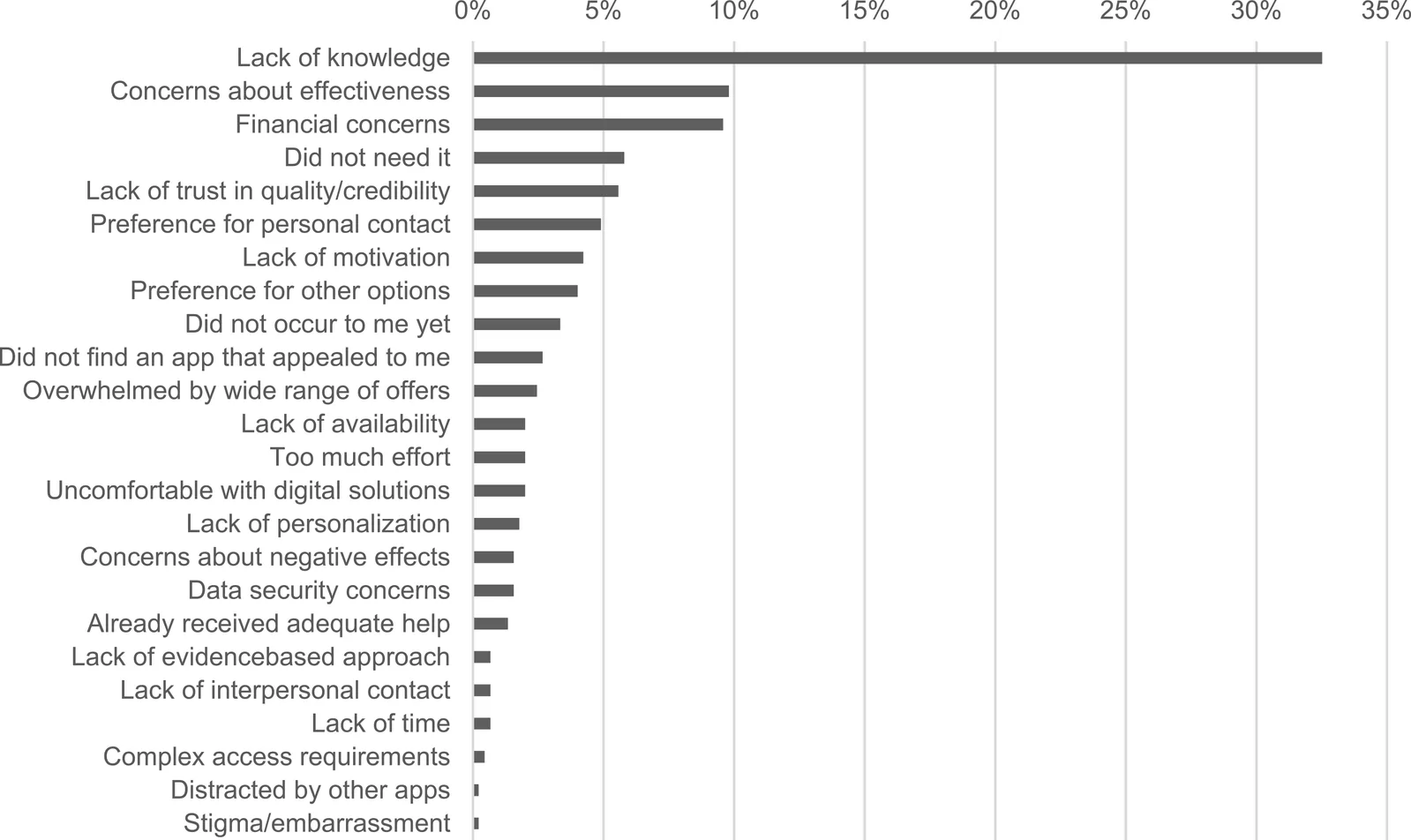
Barriers to using mental-health apps in the sample of young women (*N* = 305)

Among participants who never used mental health apps, 85.5% reported that they would be willing to use them. An open question regarding barriers to mental health app use showed that the lack of knowledge regarding available MHA and their options (32.5%), concerns about the effectiveness (9.8%), and financial concerns (9.6%) were the most potential barriers (see Fig. 2).

### Associations between psychological distress and MHA use

MHA use (encompassing both lifetime and current use) did not differ between participants with and without self-reported diagnosed lifetime mental disorder (33.2% vs. 29.5%, OR = 1.19 [0.82–1.73], *p* = .361). However, the probability of app use increased with higher values of psychological distress (K-10 score) (OR = 1.03 [1.01–1.06], *p* = .012). Associations between different expressions of psychological distress and any mental health app use are shown in Table 3. The experience of anxiety and appetite/weight problems were the most consistent predictors of app use (ORs between 1.95 and 1.99, *p*s ≤ .004).

**Table 3:** Associations between symptoms of psychological distress and mental health app use

	Separate models			Simultaneous model		
	OR	95% CI	*p*	OR	95% CI	*p*
Tension	1.77	1.13–2.78	.013*	1.46	0.90–2.37	.126
Anxiety	2.02	1.33–3.06	.001***	1.99	1.25–3.16	.004**
Depressed mood	0.91	0.63–1.33	.628	0.85	0.55–1.33	.479
Sadness	0.85	0.59–1.24	.399	0.68	0.44–1.05	.081
Irritability	1.21	0.82–1.78	.334	1.06	0.69–1.62	.801
Headaches	1.12	0.77–1.63	.545	0.91	0.60–1.37	.639
Fatigue/Exhaustion	0.88	0.56–1.38	.572	0.79	0.48–1.29	.340
Worries/Brooding	1.15	0.72–1.82	.561	0.91	0.53–1.54	.719
Social conflicts	1.43	0.94–2.17	.099	1.27	0.79–2.04	.331
Social withdrawal	1.30	0.89–1.89	.169	1.16	0.77–1.74	.488
Sleeping problems	1.27	0.88–1.84	.204	1.00	0.66–1.52	.995
Appetite or weight problems	1.96	1.33–2.90	.001***	1.95	1.27–2.99	.002**
Stomach/intestinal problems	1.45	0.99–2.11	.055	1.23	0.82–1.85	.313
Concentration problems	1.25	0.86–1.81	.239	1.07	0.70–1.64	.748
Substance use	1.32	0.72–2.44	.371	1.13	0.59–2.17	.715

*OR*  Odds ratio, *CI*  Confidence interval. In separate models, one predictor was entered at a time in logistic regressions; the simultaneous model includes all listed predictors entered at once. * *p* ≤ .05; ** *p* ≤ .01; *** *p* ≤ .001

### Results of the thematic analysis regarding attitudes towards mental health apps

Table 4 summarizes the results of the thematic analysis regarding the question by which criteria young women evaluate mental health applications as professional. A total of 346 of the participants (66.4%) responded to this question.

**Table 4:** Results of a thematic analysis of answers by 346 young women: How do you recognize professional appearance in a mental health app?

Theme	∑	Sample Quotes
*Usability Features*		
User-friendly layout	42 (12.3%)	Aesthetic and user-friendly interface; Clear and comprehensible structures
No advertisement	15 (4.4%)	Nothing is imposed or sold to you; No annoying interruptions due to advertisement
Language	13 (3.8%)	No spelling errors; Good spelling and punctuation
Reasonably selected content	11 (3.2%)	Professionally selected topics; Content has to make sense; No healing promises; No cliché phrases
Personalization	4 (1.2%)	Tailored themes; Customized apps tailored to the individually processed information
*Security Features*		
Development by professionals	50 (14.6%)	The app should be developed and tested by therapists and doctors; Collaborations with renowned experts in the given field
Evidence base	40 (11.7%)	Conforming to scientific standards; Provided information are empirically supported by scientific evidence
Cooperation with professionals	24 (7.0%)	By collaborations with universities & colleges or clinics; Ideally it runs in cooperation with a therapeutic organization
Legal notice	21 (6.1%)	Legal notice presented; Good and comprehensible legal notice
Contact opportunities	18 (5.3%)	Clearly indicate who is behind the app; Contact person within the country and headquarters
Transparency	13 (3.8%)	Who are the providers? What is the opinion of experts? Who are the users?; Application limitations clearly explained
Data protection / privacy	13 (3.8%)	Anonymity should be ensured; No need to enter irrelevant data
*Other Features*		
Reputation	48 (14.1%)	Frequently recommended, also endorsed by professional experts; Reviews from other users; Well-known; Download frequency
Fair financial deal	18 (5.3%)	No hidden costs; Ensuring access for individuals from any social background
Literature references	18 (5.3%)	Elaboration of content including citations and sources; Literature references and working with academic sources
*Miscellaneous*	4 (1.2%)	The app gets updated regularly; Questions are taken seriously; Country of origin; If it is an offer and does not force me into anything or ignites competition among those affected

Table 5 summarizes the results of the thematic analysis regarding the question what the most helpful mental health app would offer. A total of 461 participants (88.5%) responded to this question.

**Table 5:** Results of a thematic analysis of answers by 461 young women: What would the most helpful mental health app offer?

Theme	∑	Sample Quotes
*Usability Features*		
Low threshold access	37 (8.0%)	Free or financed through health insurance; No subscription; Flexible cancellations; Possible use without Wi-Fi
Personalization	34 (7.4%)	A tailored program for the user; Addressing the user’s personality. Perhaps a brief survey beforehand to gauge prior knowledge and identify goals
User-friendly layout	26 (5.6%)	Make it more appealing so I will not forget about it and feel like using it; Short sentences and appealing material
*Security Features*		
Data protection / privacy	10 (2.2%)	Anonymity; Password protection; It should be trustworthy; Safe space atmosphere
Expert approval	4 (0.9%)	Approvement by health care services; Tips which are scientifically approved
*Personal Support*		
Professional guidance	77 (16.7%)	Options to contact psychologists or psychotherapists; Qualified people who are all time available via chat, phone or in person
Peer-to-peer support	48 (10.4%)	It should provide the opportunity to anonymously exchange thoughts with others, whilst ensuring respectful interactions; Exchange with others via virtual support groups
*Technical Features*		
Reminders	22 (4.8%)	Occasional reminders to exercise and offer a reward system to stay on track; Daily reminders would help to motivate me to stay on track so I can change my behaviour
Progress monitoring	13 (2.8%)	See statistics on how one’s well-being changes over the weeks; To see progress and how much one has already worked on one’s mental health
Diagnostics	7 (1.5%)	Reliable self-diagnostics; Questionnaire to assess the current problem
Distraction management	3 (0.7%)	Other apps should be blocked while I am using the app; Teaching us to disconnect, including turning off our phones consciously for a few hours.
*Other Features*		
Adequate motivation	13 (2.9%)	Motivational and empathetic implementation; It shouldn’t put me under pressure; Does not create an unrealistic image and over-motivates to the point where one feels bad if unable to achieve some of it
*Content Features*		
Supportive / self-help interventions and exercises	60 (13.0%)	Showing ways to get out of one's problems; Self-help; Wide selection and mixture of exercises
Assistance in crisis situations	38 (8.2%)	Providing an SOS box, to document personal first aid measures and being able to quickly access this box; Capturing early signs of a potentially emerging crisis; Clear instructions during emergency situations
Navigation through health care system	36 (7.8%)	Suggestions how to find a therapist; List of psychologists nearby, especially those with available capacity
Psychoeducation	20 (4.3%)	Information on mental illness or affective disorders; Information for relatives
Self-reflection	6 (1.3%)	It helps me to question my own actions; Regular self-reflection
*Miscellaneous*	5 (1.0%)	Tracking the menstrual cycle where one can record data such as mood, hunger, etc., to see how it might be related; Possibility to listen to music whilst using the app; Convey the feeling of being normal; Recall positive things that one may have previously entered; Not providing the option to mention negative things to focus on the positive

## Discussion

The current study analysed a convenience sample of young women with an age between 18 and 35 years. The participants showed high levels of psychological distress, with fatigue, worrying, and tension as the most prevalent symptoms. The most frequently used support sources included self-help books, primary care, and psychotherapy. Although most young women expressed general interest in using MHA, only one-third reported having ever used one. Unlike self-reported diagnosed mental disorder, overall psychological distress, anxiety and appetite/weight problems were associated with having used MHA at some point in their lifetime, suggesting that subjective distress rather than formal diagnosis may drive help-seeking through MHA. This is consistent with Andersen’s distinction between perceived and professionally evaluated need [12], indicating MHA use may have been driven by perceived rather than evaluated need in this sample. The discrepancy between interest and actual use aligns with findings from a meta-analysis, incorporating results from studies on adults with mental disorders, showing that, while many consider MHA useful and express willingness to use them, only a minority actually uses them [18]. This gap between interest and use mirrors UTAUT’s distinction between use intention and actual use behavior [13]. The most common reason for non-use was a lack of knowledge about available MHA and their options, reported by about one-third of participants. Other barriers, such as concerns about effectiveness, financial or structural obstacles, mirror barriers found in adult populations seeking digital mental health support [19]. Lack of knowledge represents a predisposing factor in Andersen’s model [12], while effectiveness concerns and financial/structural obstacles correspond to the usefulness and cost dimensions of UTAUT [13], respectively. These findings may point to increasing the visibility of evidence-based MHA as a potentially relevant lever for uptake, which future research should examine. Future dissemination efforts should therefore focus on educating persons in need about opportunities and limitations of MHA, especially for young women reluctant to seek in-person consulting or psychotherapy. The need for payment or subscription fees indeed constitutes a barrier to MHA usage, particularly for younger people or those with lower income. In Germany, a subset of certified digital health interventions, including MHA, are reimbursable through statutory health insurance, which may represent an enabling factor [12] that may lower the cost barrier identified in UTAUT [13], for eligible individuals. Awareness of different access pathways may represent an important dimension of mental health literacy that future dissemination approaches should address. The finding that subjective distress and specific symptom experiences rather than formal diagnosis was associated with MHA use indicates that they currently reach a group whose needs may precede or bypass formal diagnostic thresholds. MHA may thus function as a low-threshold entry point into care for young women who experience distress but do not (yet) meet, or do not seek confirmation of, diagnostic criteria. Clinician recommendations tailored to specific symptom profiles rather than diagnostic categories may represent a promising approach worth investigating in future research. For instance, proactively introducing apps to young women presenting with anxiety or eating-related concerns could be explored as a potential low-threshold entry point into stepped care. Further, MHA should integrate clear guidance on when and how to seek professional help. Beyond that, it should be taken into account that the relationship between symptom severity and MHA usage can be non-linear in a way that higher symptom severity may motivate usage up to a point, but those with the most severe symptoms may be less engaged [20]. Further, addressing concerns about effectiveness requires transparent communication of the evidence base directly within dissemination channels, as scientific evaluation alone may not translate into perceived credibility among potential users. As many mental health professionals report insufficient knowledge about digital mental health interventions as a relevant barrier to make informed recommendations [21], they should be educated about research-based health app quality assessment frameworks [22]. As clinician recommendations reflect the social endorsement dimension of UTAUT [13], insufficient provider knowledge may limit this pathway to MHA uptake. Educational initiatives within schools, universities, and youth health services may further improve digital health literacy and awareness of professional MHA.

The young women indicated that professionalism and helpfulness in MHA are multifaceted, encompassing aspects of usability, security, and content. Helpfulness was associated with features such as personal support, technical functionality, and relevant content. These findings are consistent with recommendations for digital health interventions that highlight personalization, credibility, and usability [23]. For developers, this suggests that intuitive design, robust data security, and professionally backed content may be important considerations. Features supporting self-management, providing guidance, and offering educational resources may further enhance perceived usefulness. Beyond that, co-design with target users [24] should be integrated throughout development and evaluation of mental health apps to help improve acceptability and effectiveness. At the same time, some of the features participants desired most involve implementation challenges: Integrating professional support considerably increases operating costs and limits scalability. Peer-to-peer interaction features require continuous moderation to prevent misinformation or hostile comments [25]. Personalization presupposes the collection and processing of sensitive health data, heightening privacy and data protection requirements, which is a critical consideration given that privacy concerns were themselves reported as a barrier. User preferences should therefore not be translated directly into design specifications, rather desirability needs to be balanced against clinical safety, regulatory and data protection requirements, and economic feasibility. This tension underscores the need for user-centered but clinically supervised development processes in which users, clinicians, and developers are involved iteratively. Best-practice guidance in MHA development emphasizes structured frameworks that translate theory into design, such as the *Digital Intervention Development* Guide’s two-phase approach linking theoretical intervention techniques to specific technological features [26], together with co-production of apps with end users and stakeholders, which is increasingly considered the gold standard for ensuring acceptability and engagement [27]. At the population level, best practice for dissemination calls for addressing persistent quality problems in the marketplace, such as limited regulation and unclear data practices [28]. Within care settings, implementation is best supported through phase-matched strategies that embed digital interventions into existing clinical workflows and plan for sustainment from the outset [29].

Several limitations should be considered. Recruitment through social media platforms and a university mailing list may have introduced selection bias, as these channels predominantly reach individuals with higher digital affinity and educational attainment, which limits the generalisability of findings to the broader population. As the exclusively female sample limits the generalisability of the findings to young men, future studies should prioritise gender-balanced recruitment strategies to allow for systematic examination of potential gender differences. Moreover, thematic analysis, while providing rich insights, is subjective and dependent on researcher interpretation. Triangulating these findings with quantitative data or alternative qualitative methods could strengthen validity. In future research, longitudinal studies examining how fluctuations in distress influence MHA uptake and sustained use would be beneficial. Further studies should also investigate how symptom-specific motivations interact with design features to promote uptake and efficacy. For such studies, it would be important to assess use behaviours in addition to self-reports. Disentangling attrition from need-driven disengagement, and examining the circumstances under which use is continued or ended, are promising questions for future longitudinal research combining symptom and usage data.

## Conclusions

MHA hold promise for reducing the mental health burden among young women—a group with high need and motivation to use such tools—yet uptake and sustained utilisation remain key challenges that substantially limit their overall impact in this group. These challenges may be overcome by making young women aware of credible, well-evaluated MHA by designing apps that are genuinely usable, personalised, and responsive to users’ needs; by ensuring robust data security and professional credibility to build and maintain trust; and by embedding app use within existing health care pathways so that engagement is supported and sustained over time. Taken together, the findings suggest that realising the potential of MHA for young women requires not only evidence of effectiveness, but careful attention to how these tools are evaluated, implemented, and integrated into everyday care.

## Supplementary Information

Supplementary Material 1.

## Data Availability

The datasets used and/or analysed during the current study are available from the corresponding author upon reasonable request.

## Abbreviations

MHA: Mental Health Apps
OR: Odds Ratio
M: Mean
SD: Standard Deviation
CI: Confidence Interval
