# Supplementary material for: Barriers and facilitators of mental health app use among young women – a multimethod study
Source: BMC Womens Health. 2026 Sep 26;26:477. doi: 10.1186/s12905-026-04934-w (PMC13615588; doi:10.1186/s12905-026-04934-w)
Supplement: Supplementary file 1 [file 12905_2026_4934_MOESM1_ESM.docx]

**Additional file 1**

**Table S1.** *Correlations with Confidence Intervals*

| Variable | 1 | 2 | 3 | 4 | 5 | 6 | 7 | 8 | 9 | 10 | 11 | 12 | 13 | 14 |
| --- | --- | --- | --- | --- | --- | --- | --- | --- | --- | --- | --- | --- | --- | --- |
|  |  |  |  |  |  |  |  |  |  |  |  |  |  |  |
| 1. Tension |  |  |  |  |  |  |  |  |  |  |  |  |  |  |
|  |  |  |  |  |  |  |  |  |  |  |  |  |  |  |
| 2. Anxiety | .20** |  |  |  |  |  |  |  |  |  |  |  |  |  |
|  | [.12, .28] |  |  |  |  |  |  |  |  |  |  |  |  |  |
|  |  |  |  |  |  |  |  |  |  |  |  |  |  |  |
| 3. Depressed mood | .06 | .06 |  |  |  |  |  |  |  |  |  |  |  |  |
|  | [-.02, .15] | [-.02, .15] |  |  |  |  |  |  |  |  |  |  |  |  |
|  |  |  |  |  |  |  |  |  |  |  |  |  |  |  |
| 4. Sadness | -.01 | .17** | .32** |  |  |  |  |  |  |  |  |  |  |  |
|  | [-.10, .07] | [.09, .25] | [.24, .39] |  |  |  |  |  |  |  |  |  |  |  |
|  |  |  |  |  |  |  |  |  |  |  |  |  |  |  |
| 5. Irritability | .10* | -.02 | .04 | -.02 |  |  |  |  |  |  |  |  |  |  |
|  | [.02, .19] | [-.11, .06] | [-.05, .12] | [-.10, .07] |  |  |  |  |  |  |  |  |  |  |
|  |  |  |  |  |  |  |  |  |  |  |  |  |  |  |
| 6. Headaches | .14** | .11* | .01 | .04 | .16** |  |  |  |  |  |  |  |  |  |
|  | [.05, .22] | [.02, .19] | [-.07, .10] | [-.05, .12] | [.08, .24] |  |  |  |  |  |  |  |  |  |
|  |  |  |  |  |  |  |  |  |  |  |  |  |  |  |
| 7. Fatigue/exhaustion | .08 | .06 | .26** | .15** | .10* | .15** |  |  |  |  |  |  |  |  |
|  | [-.00, .17] | [-.03, .15] | [.18, .34] | [.07, .24] | [.02, .19] | [.06, .23] |  |  |  |  |  |  |  |  |
|  |  |  |  |  |  |  |  |  |  |  |  |  |  |  |
| 8. Worries/brooding | .20** | .29** | .21** | .20** | .01 | .06 | .07 |  |  |  |  |  |  |  |
|  | [.12, .28] | [.21, .37] | [.12, .29] | [.12, .29] | [-.07, .10] | [-.03, .14] | [-.02, .15] |  |  |  |  |  |  |  |
|  |  |  |  |  |  |  |  |  |  |  |  |  |  |  |
| 9. Social conflicts | .06 | .03 | .08 | .08 | .28** | .15** | .11** | -.03 |  |  |  |  |  |  |
|  | [-.02, .15] | [-.05, .12] | [-.01, .17] | [-.00, .17] | [.20, .36] | [.07, .23] | [.03, .20] | [-.12, .05] |  |  |  |  |  |  |
|  |  |  |  |  |  |  |  |  |  |  |  |  |  |  |
| 10. Social withdrawal | .06 | .09* | .24** | .08 | .06 | .08 | .10* | .11* | .12** |  |  |  |  |  |
|  | [-.02, .15] | [.00, .17] | [.15, .32] | [-.00, .17] | [-.02, .15] | [-.01, .16] | [.02, .19] | [.02, .19] | [.03, .20] |  |  |  |  |  |
|  |  |  |  |  |  |  |  |  |  |  |  |  |  |  |
| 11. Sleeping problems | .12** | .17** | .13** | .12** | .11* | .15** | .16** | .19** | .14** | .11* |  |  |  |  |
|  | [.04, .21] | [.09, .26] | [.04, .21] | [.04, .21] | [.02, .19] | [.06, .23] | [.07, .24] | [.11, .27] | [.06, .22] | [.03, .20] |  |  |  |  |
|  |  |  |  |  |  |  |  |  |  |  |  |  |  |  |
| 12. Appetite/weight problems | .11* | .07 | .16** | .20** | .09 | .09* | .10* | .06 | .11* | .15** | .20** |  |  |  |
|  | [.02, .19] | [-.02, .15] | [.08, .25] | [.11, .28] | [-.00, .17] | [.01, .18] | [.01, .18] | [-.03, .14] | [.03, .20] | [.07, .23] | [.11, .28] |  |  |  |
|  |  |  |  |  |  |  |  |  |  |  |  |  |  |  |
| 13. Stomach/intestinal problems | .05 | .10* | -.04 | .07 | .09 | .14** | .03 | .02 | .14** | .08 | .14** | .14** |  |  |
|  | [-.04, .13] | [.02, .19] | [-.13, .04] | [-.02, .15] | [-.00, .17] | [.05, .22] | [-.06, .11] | [-.07, .10] | [.06, .23] | [-.00, .17] | [.06, .23] | [.06, .23] |  |  |
|  |  |  |  |  |  |  |  |  |  |  |  |  |  |  |
| 14. Concentration problems | .09* | .19** | .23** | .15** | .10* | .14** | .15** | .21** | .17** | .14** | .16** | .18** | -.07 |  |
|  | [.00, .17] | [.10, .27] | [.15, .31] | [.07, .23] | [.02, .19] | [.06, .23] | [.06, .23] | [.12, .29] | [.08, .25] | [.05, .22] | [.07, .24] | [.09, .26] | [-.16, .02] |  |
|  |  |  |  |  |  |  |  |  |  |  |  |  |  |  |
| 15. Substance use | .06 | .06 | .10* | .08 | .11** | .05 | .08 | .02 | .15** | .02 | .13** | .08 | .05 | .08 |
|  | [-.03, .14] | [-.03, .14] | [.01, .18] | [-.00, .17] | [.03, .20] | [-.04, .14] | [-.00, .17] | [-.06, .11] | [.07, .24] | [-.06, .11] | [.05, .22] | [-.00, .17] | [-.04, .13] | [-.00, .17] |
|  |  |  |  |  |  |  |  |  |  |  |  |  |  |  |

*Notes.* Values in square brackets indicate the 95% confidence interval for each correlation. * indicates *p* ≤ .05. ** indicates *p* ≤ .01.
